# A role for β‐catenin in diet‐induced skeletal muscle insulin resistance

**DOI:** 10.14814/phy2.15536

**Published:** 2023-02-17

**Authors:** Stewart W. C. Masson, Waruni C. Dissanayake, Sophie C. Broome, Christopher P. Hedges, Wouter M. Peeters, Martin Gram, David S. Rowlands, Peter R. Shepherd, Troy L. Merry

**Affiliations:** ^1^ Discipline of Nutrition, Faculty of Medical and Health Sciences The University of Auckland Auckland New Zealand; ^2^ Maurice Wilkins Centre for Molecular Biodiscovery The University of Auckland Auckland New Zealand; ^3^ Department of Molecular Medicine and Pathology, Faculty of Medical and Health Sciences The University of Auckland Auckland New Zealand; ^4^ School of Sport, Exercise and Nutrition Massey University Auckland New Zealand; ^5^ Faculty of Medical Sciences Newcastle University Newcastle UK

**Keywords:** glucose transport, insulin resistance, obesity, Wnt‐signaling

## Abstract

A central characteristic of insulin resistance is the impaired ability for insulin to stimulate glucose uptake into skeletal muscle. While insulin resistance can occur distal to the canonical insulin receptor‐PI3k‐Akt signaling pathway, the signaling intermediates involved in the dysfunction are yet to be fully elucidated. β‐catenin is an emerging distal regulator of skeletal muscle and adipocyte insulin‐stimulated GLUT4 trafficking. Here, we investigate its role in skeletal muscle insulin resistance. Short‐term (5‐week) high‐fat diet (HFD) decreased skeletal muscle β‐catenin protein expression 27% (*p* = 0.03), and perturbed insulin‐stimulated β‐catenin^S552^ phosphorylation 21% (*p* = 0.009) without affecting insulin‐stimulated Akt phosphorylation relative to chow‐fed controls. Under chow conditions, mice with muscle‐specific β‐catenin deletion had impaired insulin responsiveness, whereas under HFD, both mice exhibited similar levels of insulin resistance (interaction effect of genotype × diet *p* < 0.05). Treatment of L6‐GLUT4‐myc myocytes with palmitate lower β‐catenin protein expression by 75% (*p* = 0.02), and attenuated insulin‐stimulated β‐catenin phosphorylation^S552^ and actin remodeling (interaction effect of insulin × palmitate *p* < 0.05). Finally, β‐catenin^S552^ phosphorylation was 45% lower in muscle biopsies from men with type 2 diabetes while total β‐catenin expression was unchanged. These findings suggest that β‐catenin dysfunction is associated with the development of insulin resistance.

## Graphical abstract

β‐catenin is an emerging regulator of insulin‐stimulated glucose transport. Here, we demonstrate lower β‐catenin protein abundance in insulin resistant mice, and perturbed β‐catenin phosphorylation in men with type 2 diabetes.

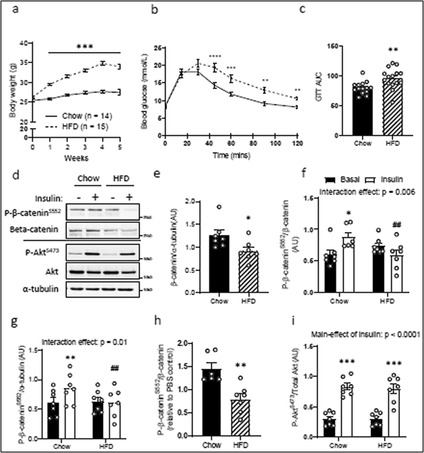

## 1 INTRODUCTION

Insulin resistance (IR) is the progressive failure of skeletal muscle, adipose tissue, and the liver to respond to insulin, and is a major risk factor for type 2 diabetes (Goldstein, 2002; James et al., 2021; Meigs et al., 2007; Sung et al., 2012). A key characteristic of IR is impaired insulin‐stimulated skeletal muscle glucose uptake (DeFronzo, 1988; Sylow et al., 2021). Despite this impairment, insulin‐resistant individuals often exhibit normal fasting blood glucose concentrations, due to compensatory hyperinsulinemia. In healthy muscle, insulin promotes the translocation of the insulin‐responsive glucose transporter (GLUT4) to the plasma membrane, via the insulin receptor‐PI3k‐Akt signaling pathway (Klip et al., 2014; Sylow et al., 2021). Once docked, GLUT4 facilitates transport of glucose into the cell. Interestingly, during the early stages of IR, skeletal muscle insulin‐stimulated glucose uptake is impaired, despite proximal insulin signaling being maintained (Goldstein, 2002; Hoehn et al., 2008; Kim et al., 1999; Krook et al., 2000). This suggests that the initial site of dysfunction may be downstream of the canonical insulin receptor‐PI3k‐Akt signaling pathway.

Recently, we identified the structural protein β‐catenin as a regulator of skeletal muscle glucose uptake (Masson et al., 2020; Masson et al., 2021). Insulin promotes the phosphorylation of β‐catenin^S552^, which in turn regulates GLUT4‐containing vesicle movement via changes in the actin cytoskeleton. Cross‐species gene expression analysis has identified β‐catenin as a possible central node in the development of IR based on its predicted interactions with many of the proteins involved in the insulin signaling pathway (Chaudhuri et al., 2015). Furthermore, β‐catenin has been implicated in type 2 diabetes through its known interaction with the diabetes risk gene, *Tcf7l2* (Cauchi et al., 2006; Chandak et al., 2007; Chen et al., 2018; Ferreira et al., 2018; Jin, 2016; Wang et al., 2015). However, it is unknown whether β‐catenin becomes dysfunctional in skeletal muscle during the development of IR. Here, we investigated how β‐catenin expression and signaling changes under conditions of insulin resistance. We report that in both in vivo and in vitro models of insulin resistance, including men with type 2 diabetes, β‐catenin signaling is perturbed. Furthermore, in mice fed a high‐fat diet and palmitate‐treated cells, β‐catenin protein expression is attenuated. We propose that β‐catenin dysfunction is an early defect in the development of skeletal muscle insulin resistance.

## 2 METHODS

All reagents, unless otherwise stated, were purchased from Sigma‐Aldrich Chemicals. Antibodies are given in Table S1.

### 2.1 Murine breeding and housing conditions

Inducible muscle‐specific β‐catenin‐deficient mice were generated by crossing β‐catenin^flox^ (B6.129‐Ctnnb1^tm2Kem^/KnwJ), and HSA‐MCM Cre (Tg(ACTA1‐cre/Esr1*)2Kesr/J) mice (obtained from Jackson Laboratories, USA, stock numbers #0044152 and #025750) resulting in β‐catenin^lox/lox;CreMCM/+^ (Brault et al., 2001; McCarthy et al., 2012), which are referred to as BCAT‐mKO, and we have recently described these mice in detail (Masson et al., 2020). β‐catenin^lox/lox;CreMCM/−^ littermates were used as control (WT) mice. Cre expression was induced at 8–12 weeks of age by administration of 2 mg tamoxifen (5% ethanol, sunflower oil; Cayman Chemicals) via oral gavage for five consecutive days. WT and BCAT‐mKO mice received the same dose of tamoxifen. C57BL/6j were breed in‐house. All mice were maintained in groups of 4–10 per cage at 23°C, in a temperature‐controlled animal facility with 12‐h light–dark cycle and ad libitum access to water and a standard rodent chow diet (Teklad TB 2018; Harlan, Madison, WI, USA) or a high‐fat diet (SF04‐027; Specialty Feeds, Australia) from 15–19 weeks of age. The number of animals per cage was evenly spread between experimental groups and based on littermates to avoid conflict between mice. Only male mice were used in these experiments as we were unable to achieve β‐catenin knockdown in female BCAT‐mKO mice. All experiments were approved by the University of Auckland animal ethics committee, Auckland, New Zealand.

### 2.2 Mouse metabolic experiments

For high‐fat diet experiments, male C57BL/6j and WT or BCAT‐mKO mice were fed either chow or a high‐fat diet for 5 weeks prior to experimentation. This duration of diet was chosen to capture the early defects in insulin resistance. For metabolic cage experiments, mice were single housed at 23°C. Food and water intake, energy expenditure, and respiratory exchange ratio was determined using a Promethion High‐Definition Multiplexed Respirometry System (Sable Systems International) for 1 week, and the final 48 h were used for analysis. For glucose and insulin tolerance tests, mice were fasted for 4–6 h, then administered either an oral gavage of 1 mg/g glucose, or an intraperitoneal injection of 0.7 mU/g Actrapid insulin (Novo Nordisk), respectively. Blood glucose measures were taken at *t* = 0, 15, 30, 45, 60, 90, and 120 min using a handheld glucose meter (Accu‐chek Performa; Roche, Basal, Switzerland). Plasma insulin was determined using an Ultra‐Sensitive Mouse Insulin ELISA kit (Crystal Chem) according to manufacturer's instructions. Plasma NEFA was determined using HR‐2 color reagent (Wako Chemicals) adapted to microplate format. Briefly, 2.5 μl of standard/samples was mixed with 100 μl of color reagent A, and incubated at 37°C for 10 min, followed by addition of 50 μl color reagent B and incubation at 37°C for 15 min. Absorbances were determined as per manufacturer's protocol. For in vivo protein analysis BCAT‐mKO, WT, or C57BL/6j mice were fasted for 6 h (0800–1400 h) prior to intraperitoneal injections of either saline or insulin (1 mU/g). Mice were killed by cervical dislocation 10 min posttreatment and tissues were rapidly dissected. All tissues were snap‐frozen in liquid nitrogen for further analysis.

### 2.3 In vivo glucose uptake

In vivo glucose uptake was determined as previously described (Lundell et al., 2019), 21–25‐week‐old BCAT‐mKO and WT mice were fasted for 6 h (0800–1400 h) prior to intraperitoneal injection with radiolabelled 2‐[2,6‐3H]‐2 deoxy‐d‐glucose, specific activity 0.128 μCi ml^−1^, (100 μl of PBS/animal, 1 mCi/ml) and either 1 mU/g of insulin or a PBS control. After 30 min mice were culled, and tissues were quickly washed in ice‐cold PBS and snap‐frozen in liquid nitrogen. Quantification of uptake was determined as previously described (Campbell & Febbraio, 2002). A portion of tissue was lysed in 1 M NaOH followed by neutralization in 1 M HCl. Lysate was then deproteinised in perchloric acid (to yield total 2‐DG) and equal volumes of BaOH and ZnSO_4_ (to yield unphosphorylated 2‐DG). The difference (phosphorylated 2‐DG) was then expressed relative to brain‐phosphorylated 2‐DG and insulin/PBS fold changes were compared against previously published data from chow‐fed mice (Masson et al., 2020; Masson et al., 2021).

### 2.4 Cell culture and palmitate experimentation

L6‐Glut4‐myc myoblasts (Kerafast) were grown in low glucose DMEM supplemented with 10% fetal bovine serum and 1% penicillin/streptomycin. Differentiation was induced by changing media to low glucose DMEM supplemented with 2% horse serum for 2–3 days. For palmitate experiments, L6‐Glut4‐myc myotubes were incubated overnight in either DMEM + BSA‐PA conjugate or DMEM + BSA. Palmitic acid was conjugated to BSA by first dissolving it in 50°C ethanol prior to dilution in low glucose DMEM containing 2% BSA. Vehicle controls were low glucose DMEM with 2% BSA and equivalent volumes of EtOH.

### 2.5 Actin polymerization assay

Determination of F‐G actin ratio was performed as previously described (Sorrenson et al., 2016). Briefly, L6‐G4‐myc myotubes were stimulated with 100 nM of insulin and lysed in 37°C actin stabilization buffer (50 mM PIPES, pH 6.9, 50 mM NaCl, 5 mM MgCl2, 5 mM EGTA, 5% (v/v) glycerol, 0.1% nonidet P‐40, 0.1% Triton X‐100, 0.1% Tween 20, 0.1% 2‐mercaptoethanol, 0.001% antifoam A, 1 mM ATP, and protease inhibitors). Lysates were centrifuged for 5 min, 2000*g*, at 37°C, the supernatant was then recovered and centrifuged for 1 h, 100,000*g* at 37°C. The pellet (F‐actin) was resuspended in Milli‐Q water containing 10 μM cytochalasin D. Both the pellet fraction and the supernatant (G‐actin containing) were analyzed by immunoblotting. Analysis was performed by calculating F‐actin as a percentage of total (sum of F and G actin).

### 2.6 Immunoblotting

Immunoblotting was performed as previously described (Merry et al., 2020). For tissue, snap‐frozen muscle was minced using scissors in a lysis buffer containing Tris–HCl 20 mM, NaCl 150 mM, EDTA 1 mM, EGTA 1 mM, Triton X 1%, NP40 1%, Na_4_P_2_O_7_ 2.5 mM, β‐glycerol phosphate 1 mM, Na_3_VO_4_ 1 mM, and NaF 100 mM, and then homogenized using a Qiagen TissueLyser. For cells, ice‐cold lysis buffer was added to plates before being scraped while cells were placed on ice. In both cases, lysate was centrifuged at 4°C, 20,000*g* for 10 min. The resulting supernatants were resolved using SDS‐PAGE, following standard procedures. Membranes were incubated in primary antibody overnight at 4°C (Table S1), before being incubated in secondary antibody for 1–2 h at room temperature and imaged using chemiluminescence reagents. Antibodies were previously validated by either genetic knockdown or pharmacological inhibition (Goel et al., 2012; Masson et al., 2020; Sorrenson et al., 2016). Quantification was performed using ImageJ (National Institutes of Health). Phosphorylation of proteins was calculated as phosphorylated/total while total protein expression was determined as target/loading control.

### 2.7 In vitro glucose uptake

For in vitro glucose uptake assays, differentiated L6‐Glut4‐myc were serum starved for 3 h in DMEM containing 0.2% BSA, and then washed three times with sterile HEPES‐buffered saline (HBS; 140 mM NaCl, 20 μM HEPES, 2.5 mM MgSO4, 1 mM CaCl2, 5 mM KCl, and pH 7.4, 25°C). Cells were incubated for 10 min in HBS containing 100 mM 2‐deoxyglucose and 0.1% BSA before a 20‐min pre‐stimulation with insulin (10–100 nM). Cells were then incubated in HBS containing [3H]‐2‐deoxyglucose, 0.2% BSA and either vehicle controls or relevant treatment. After 10 min, glucose uptake was stopped by removing media and placing cells immediately on ice. Cells were lysed in 10% Triton X and read in for 1 min with a single label protocol. Glucose transport was expressed relative to respective protein concentrations, as determined by bicinchoninic acid (BCA) assay.

### 2.8 Participant recruitment

Sedentary men with non‐insulin‐dependent type 2 diabetes (39–68 y, BMI 23–38 kg/m^2^) and healthy controls (34–57 years, BMI 21–32 kg/m^2^) were recruited at medical centers and from the local community. Participants provided written consent in accordance with the protocol approved by the Central Health and Disability Ethics Committee, New Zealand 14/CEN/194.

### 2.9 Skeletal muscle biopsy and analysis

Skeletal muscle tissue (~100 mg frozen weight) from *m. vastus lateralis* was obtained using the percutaneous Bergstrom needle technique (Bergstrom, 1975) following an overnight fast. After applying local anesthesia (1% Xylocaine), a small incision was made in the skin of the left leg to access the *m. vastus lateralis*. Samples were immediately freed from any visible fat and blotted dry to remove excess blood. Muscle samples for western blotting were immediately snap‐frozen in liquid nitrogen and stored at ‐80°C until further analysis. Human muscle protein samples were extracted with modified RIPA buffer (50 mM Tris, pH 8.0, 75 mM NaCl, 0.3% NP40, 1% sodium deoxycholate, and 0.1% SDS) containing EDTA‐free protease and phosphatase inhibitors (Sigma‐Aldrich, St Louis, MO) using an automatic homogenization blender (IKA) for 1 min. Sample lysates were placed in an orbital shaker for 1 h at 4°C before centrifuging at 600*g* for 15 min at 4°C. The extracted protein was then resolved using SDS‐PAGE, as detailed above.

### 2.10 Statistical analysis

Statistical analysis was performed in GraphPad Prism 9.3 (GraphPad Software Incorporated). Area under the curve for glucose and insulin tolerance tests was calculated from 0 mM glucose using the trapezoidal method. Specific analyses are detailed in figure legends with biological replicates denoted as individual data points or elsewhere in the figure. The statistical significance level for all analyses was set to *p* < 0.05.

## 3 RESULTS

### 3.1 Short‐term HFD feeding lowers muscle β‐catenin expression and S552 phosphorylation

**FIGURE 1:**
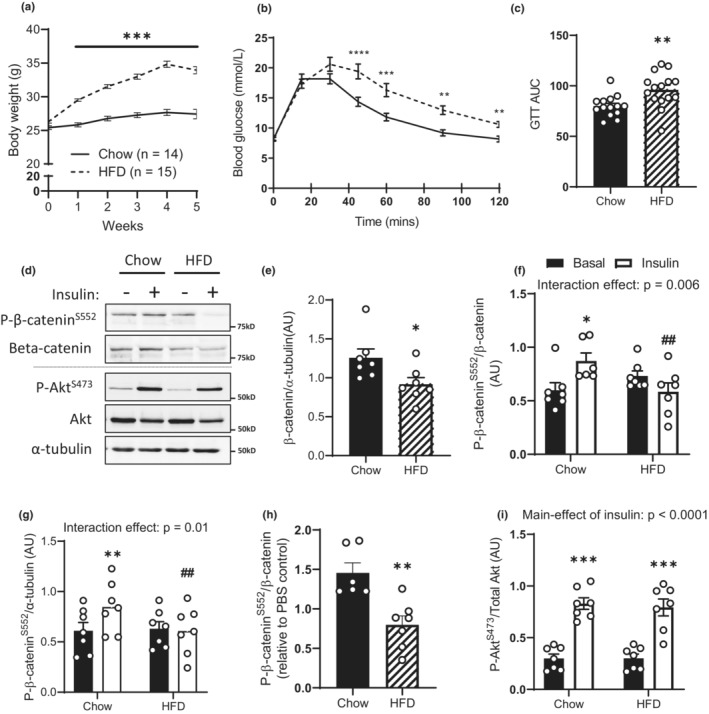
Short‐term HFD induces glucose intolerance and decreases muscle β‐catenin expression without impairing insulin‐mediated Akt signaling. Body weight (a), glucose‐tolerance (b, c), gastrocnemius β‐catenin protein expression (d, e), insulin signaling (f–h), and were determined in male C57Bl/6J mice fed either a high‐fat (HFD) or chow diet for 6 weeks. Results are mean ± SE, with *n* shown as individual data points or in figure legend. Area under the curve (AUC) was calculated in GraphPad prism using the trapezoidal method. Significance was determined by two‐way RM‐ANOVA with LSD post hoc analysis (a, b), two‐way ANOVA with LSD post hoc analysis (f, h), or Student's two‐tailed t‐test (c, e, g). *p < 0.05, **p < 0.01, ***p < 0.001 significance within groups. AUC, area under curve.

High‐fat diet fed C57Bl/6 mice gained more weight than chow‐fed control mice (main effect of diet: *p* < 0.001; Figure 1a), and after 5‐week HFD‐fed mice were glucose intolerant (main effect of diet: *p* = 0.004 for glucose tolerance test; Figure 1b,c). High‐fat diet feeding reduced skeletal muscle β‐catenin expression and attenuated insulin‐mediated phosphorylation of β‐catenin^S552^ (interaction effect of HFD vs insulin: *p* = 0.006) but not Akt^S473^ (main effect of diet: *p* = 0.75) phosphorylation (Figure 1d–i). To assess whether the apparent differences in β‐catenin^S552^ phosphorylation are simply the result of normalizing against changing β‐catenin expression, we also analyzed β‐catenin phosphorylation relative to α‐tubulin expression. Consistent with a defect in both β‐catenin expression and signaling, β‐catenin^S552^ phosphorylation is also lower HFD‐fed mice even when normalized against α‐tubulin expression (Figure 1g; interaction effect *p* = 0.01).

### 3.2 Insulin resistance due to loss of skeletal muscle β‐catenin is not exacerbated by short‐term HFD feeding

**FIGURE 2:**
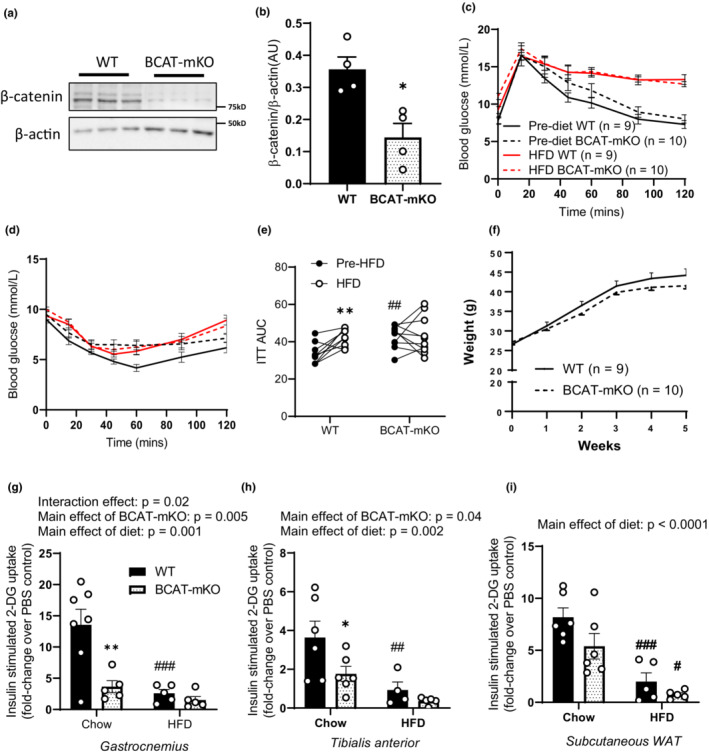
Mice lacking skeletal muscle β‐catenin are insulin resistant but are resistant to additional diet‐induced insulin‐resistance. 1 month after tamoxifen treatment, gastrocnemius muscle β‐catenin protein expression was determined by immunoblotting in male WT and BCAT‐mKO mice (a, b). Prior to 5 weeks of high‐fat diet (HFD) feeding (c) WT and BCAT‐mKO mice underwent baseline glucose (d) and insulin (e) tolerance tests. Following 5 weeks of HFD feeding, WT and BCAT‐mKO mice underwent another round of glucose and insulin tolerance tests (d–f). To assess tissue‐specific insulin resistance, in vivo glucose uptake of gastrocnemius (g), tibialis anterior (h), and subcutaneous adipose tissue (i) was then determined and compared to previous published glucose up data from chow‐fed mice (Masson et al., 2020). Results are mean ± SE, with sample size (*n*) shown as individual data points or in figure legend. Area under the curve (AUC) was calculated in GraphPad prism using the trapezoidal method. Significance was determined by Student's two‐tailed *t*‐test (b), two‐way RM‐ANOVA with LSD post hoc analysis (c–f) or two‐way ANOVA with LSD post hoc analysis (g–i). *p < 0.05, **p < 0.01 significance within groups. ^#^ *p* < 0.05, ^##^ *p* < 0.01, ^###^ *p* < 0.001 significance between groups.

We have previously shown that β‐catenin is required for optimal insulin‐stimulated glucose uptake in chow‐fed mice (Masson et al., 2020). To determine if short‐term HFD‐induced impairment in skeletal muscle β‐catenin regulation contributes to muscle insulin resistance, we utilized BCAT‐mKO mice following induction of skeletal muscle β‐catenin loss (Figure 2a,b). We hypothesized that if dysregulation of skeletal muscle β‐catenin is involved in the development of diet‐induced insulin resistance, short‐term HFD feeding would have a lesser impact on insulin responsiveness in mice lacking skeletal muscle β‐catenin. We performed glucose and insulin tolerance tests on 15–19‐week old WT and BCAT‐mKO mice, and consistent with our previous work (Masson et al., 2020), chow‐fed BCAT‐mKO had similar glucose tolerance (Figure 2c; main‐effect of KO: *p* = 0.28) but were less insulin responsive (as determined by ITT) than WT mice (main effect of KO: *p* = 0.03; Figure 2d,e). When fed a HFD WT and BCAT‐mKO mice gained a similar amount of body weight (Figure 2f), and had similar food intake, water intake, energy expenditure, substrate utilization (as assessed by respiratory exchange ratio; RER), plasma insulin, and non‐esterified fatty acid levels (Figure S1a–f). We repeated glucose and insulin tolerance tests on the same mice following 5 weeks of HFD feeding. Compared to pre‐HFD, high‐fat feeding impaired glucose tolerance (as determined by GTT) to a similar extent in both WT and BCAT‐mKO mice (main effect of diet: *p* = 0.017; Figure 2d). In contrast, HFD feeding attenuated blood glucose response to an insulin bolus (ITT) relative to pre‐HFD in WT mice, but not BCAT‐mKO mice (interaction effect between diet and genotype: *p* = 0.04; Figure 2d,e). To determine whether the impairment in insulin responsiveness associated with HFD or loss skeletal muscle β‐catenin could be explained by reduced glucose transport, we next measured insulin‐stimulated skeletal muscle and subcutaneous white adipose tissue (WAT) glucose uptake. To compare the effect of HFD feeding on WT and BCAT‐mKO in vivo glucose uptake, we determined insulin‐stimulated fold increases relative to unstimulated control mice and compared this against our previously published chow‐fed diet data (Masson et al., 2020). Consistent with ITT data, HFD feeding lowered insulin‐stimulated glucose uptake into gastrocnemius (*p* = 0.006) and tibialis anterior (*p* = 0.013) muscles of WT mice, but not BCAT‐mKO mice (Figure 2g,h). A similar HFD‐induced impairment of insulin‐stimulated glucose uptake was seen in WAT from WT and BCAT‐mKO mice (main effect of diet; *p* < 0.001; Figure 2i).

### 3.3 Canonical Wnt‐signaling is not disrupted in skeletal muscle of HFD‐fed mice

**FIGURE 3:**
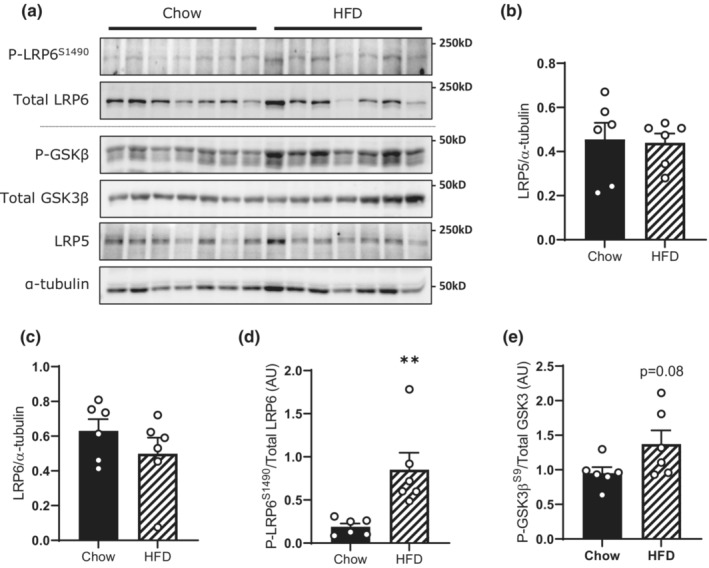
Loss of β‐catenin following short‐term HFD is not due to impaired Wnt‐signaling. Immunoblotting analysis of the Wnt‐signaling pathway from male C57Bl/6j mouse gastrocnemius muscles following 5 weeks of HFD or chow control feeding (a). Quantification of LRP5 (b), LRP6 (c), phosphorylated LRP6^S1490^ (d), and phosphorylated GSK3β^S9^ (e). Results are mean ± SE, with sample size (*n*) shown as individual data points. Significance was determined by Student's two‐tailed *t*‐test (b–e). **p < 0.01 significance between groups.

Alterations in canonical Wnt‐signaling under HFD conditions could explain the reduction in skeletal muscle β‐catenin (Figure 1d,e). To test this hypothesis, we measured phosphorylation and expression of Wnt‐signaling mediators in gastrocnemius muscles from HFD‐fed mice. High‐fat diet feeding did not affect the expression of LRP5 (Figure 3b) or LRP6 (Figure 3c) but increased the phosphorylation of LRP6 (Figure 3d) and tended (*p* = 0.08) to increase phosphorylation of GSK3β (Figure 3e). This is surprising because activation of the Wnt‐signaling pathway inhibits the β‐catenin degradation complex leading to increased β‐catenin protein expression (Ding et al., 2000; Hinck et al., 1994). This suggests that HFD‐induced loss of β‐catenin is not via disruption of canonical Wnt‐signaling.

### 3.4 Palmitic acid treatment of myocytes reduces β‐catenin expression and insulin‐induced Actin remodeling

**FIGURE 4:**
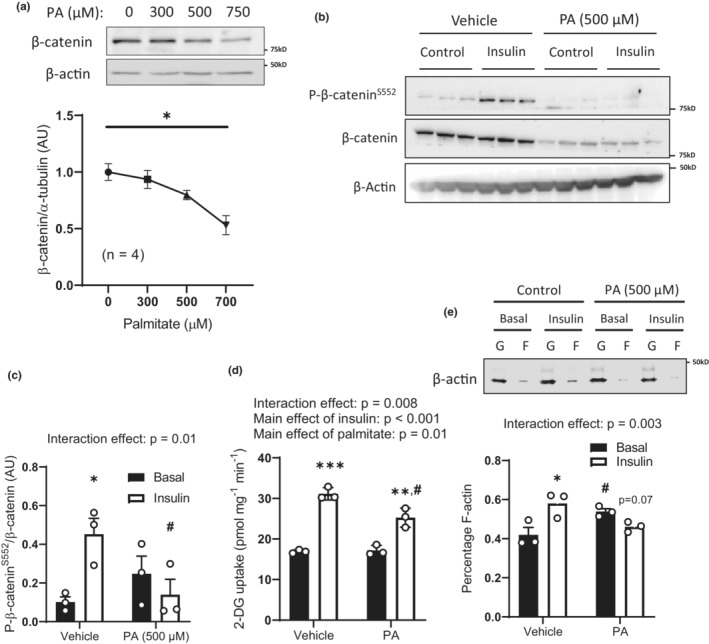
Palmitic acid treatment reduces β‐catenin and impairs insulin‐stimulated glucose uptake and actin remodeling in L6‐Glut4‐myc myotubes. L6‐Glut4‐Myc myoblasts were treated with increasing concentrations of palmitic acid (0–750 μM) and β‐catenin protein expression was determined by immunoblotting (a). Following this, L6‐Glut4‐Myc myoblasts were treated with vehicle or 500 μM palmitic acid and β‐catenin^S552^ phosphorylation (b, c), glucose uptake (d), and Actin remodeling (e) was determined in L6‐Glut4‐myc myoblasts following treatment with either insulin or vehicle. Results are mean ± SE, with sample size (*n*) shown as individual data points or in the figure legend. Significance was determined by one‐way ANOVA (a), and two‐way ANOVA with LSD post hoc analysis (c–e). *p < 0.05, **p < 0.01, ***p < 0.001 significance within groups. ^#^ *p* < 0.05, *p* significance between groups.

The proposed mechanism by which β‐catenin regulates GLUT4 translocation and glucose transport is modulation of the actin cytoskeleton (Masson et al., 2020). To investigate whether metabolic stress similarly impairs insulin's ability to promote cytoskeleton remodeling, we treated L6‐Glut4‐myc myocytes with palmitic acid. Consistent with HFD feeding of mice, we observed a palmitic acid dose‐dependent decrease in β‐catenin expression (Figure 4a,b). At dose of 500 μM, a blunting of insulin‐stimulated increases in β‐catenin^S552^, glucose uptake, and F‐actin formation dynamics were observed (Figure 4c–f).

### 3.5 Reduced β‐catenin^S552^ phosphorylation in skeletal muscle from men with type 2 diabetes

**FIGURE 5:**
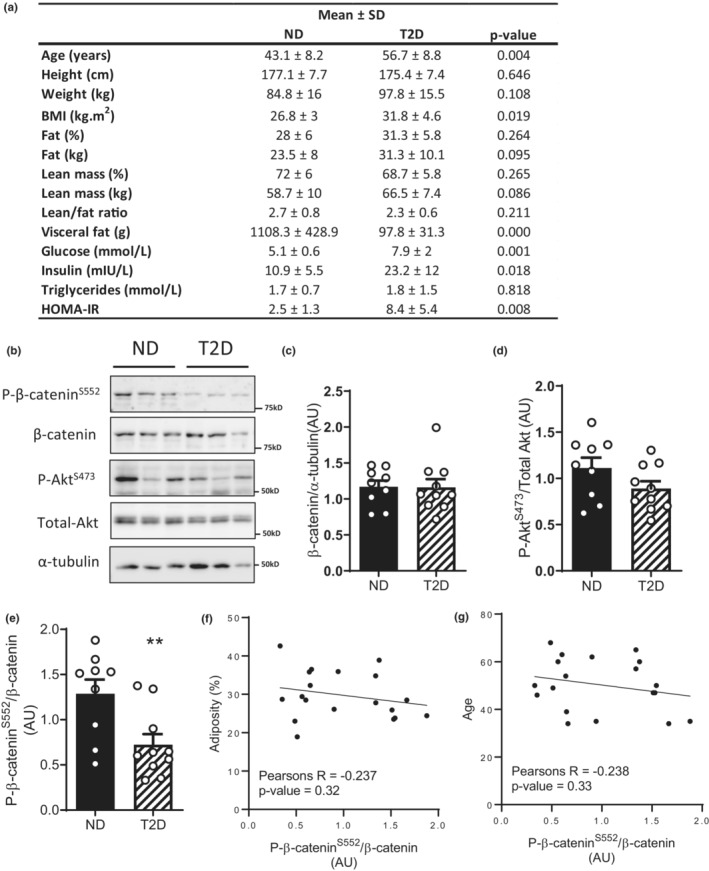
Beta‐catenin^S552^ phosphorylation is reduced in skeletal muscle of humans with type 2 diabetes. Characteristics of men with (T2D) or without (ND) type 2 diabetes participants (a). Immunoblotting (b) of biopsies taken from *m. vastus lateralis* of men with (T2D) or without type 2 diabetes (ND). Quantification of β‐catenin protein expression (c), Akt^S473^ phosphorylation (d), and β‐catenin^S552^ phosphorylation (e). Correlation analysis between β‐catenin^S552^ phosphorylation, adiposity (f), and age (g). Results are mean ± SD (a) or ± SE (c, d), with sample size (*n*) shown as individual data points. Significance was determined by Student's two‐tailed *t*‐test (a, c–e).

To determine whether our findings extend to humans, we measured β‐catenin expression, and β‐catenin^S552^ phosphorylation in skeletal muscles samples from middle‐aged men with type 2 diabetes (T2D) and non‐diabetic controls (ND). The T2D group were 14 years older than ND group (Figure 5a; *p* = 0.004), had a higher BMI (Figure 5a; *p* = 0.019), fasting blood glucose (Figure 5a; *p* = 0.001), insulin (Figure 5a; *p* = 0.018), and HOMA‐IR (Figure 5a; *p* = 0.008). Skeletal muscle β‐catenin protein expression and Akt^S473^ phosphorylation was similar in the T2D and ND groups, however, β‐catenin^S552^ phosphorylation was significantly lower in T2D muscle compared to ND (Figure 5b–e). Given the ND and T2D groups were not matched for age or BMI, we performed correlation analysis between β‐catenin^S552^ phosphorylation, age, and adiposity to test whether our effects could be mediated by age or adiposity independently of diabetes status (Figure 5f,g), neither age nor adiposity significantly correlated with β‐catenin^S552^ phosphorylation. These results provide evidence of dysregulated β‐catenin signaling in insulin‐resistant skeletal muscle.

## 4 DISCUSSION

The mechanisms underpinning skeletal muscle insulin resistance are not fully resolved, but there is evidence that impairment of insulin‐facilitated skeletal muscle glucose transport occurs distal to IR‐Akt signaling (Goldstein, 2002; Hoehn et al., 2008; Kim et al., 1999; Krook et al., 2000). Here, we provide evidence that short‐term HFD feeding of mice reduces skeletal muscle β‐catenin protein expression independently of Wnt‐signaling, and that β‐catenin^S552^ phosphorylation is perturbed in skeletal muscle from HFD‐fed mice and men with type 2 diabetes. Furthermore, mice deficient in skeletal muscle β‐catenin did not become more insulin resistant following short‐term HFD, suggesting β‐catenin dysregulation may play a role in the development of insulin resistance. Lower skeletal muscle β‐catenin in high‐fat fed is suggestive that HFD feeding drives β‐catenin loss and this contributes to diet‐induced insulin resistance. However, based on the time‐points studied, we cannot rule out that insulin resistance drives a decrease in β‐catenin abundance and function. Measurements of skeletal muscle β‐catenin levels prior to the development of insulin resistance and rescue experiments where β‐catenin overexpression is triggered in already insulin‐resistant muscle would be key to determining the direction of causality.

Previously we have shown that in skeletal muscle, insulin‐mediated β‐catenin^S552^ phosphorylation regulates actin‐cytoskeleton remodeling to facilitate GLUT4‐mediated glucose transport (Masson et al., 2020). Although loss of β‐actin itself is dispensable for optimal glucose uptake at physiological levels of insulin, there is increasing evidence that the modulation of the actin cytoskeleton is a critical step in GLUT4 translocation and that its disruption impairs insulin‐stimulated glucose transport (Brozinick Jr. et al., 2007; Khayat et al., 2000; Masson et al., 2020; Tong et al., 2001; Török et al., 2004). We interpret these findings to suggest that β‐catenin dysfunction may, in part, be involved in the development of skeletal insulin resistance and act by impairing insulin‐mediated actin‐cytoskeleton remodeling.

An important caveat in this work is the disconnect between mice and human models of insulin resistance. In HFD‐fed mice, both total β‐catenin expression and insulin‐stimulated β‐catenin^S552^ phosphorylation were lowered; however, in men with type 2 diabetes, only β‐catenin phosphorylation was reduced. While the reason for this is not clear, it may relate to the effects of diabetes medication. Metformin can suppress both β‐catenin protein expression (Park et al., 2019) and β‐catenin^S552^ phosphorylation (Amable et al., 2019) via AMPK, while insulin has been shown to activate Wnt/β‐catenin signaling and increase levels of β‐catenin protein expression (Cabrae et al., 2020). It is also important to note that the phosphorylation levels of both Akt^S473^ and β‐catenin^S552^ are taken from muscle biopsies without any form of insulin stimulation. This limits the conclusions which can be drawn regarding the ability for insulin to stimulate phosphorylation of β‐catenin under insulin‐resistant conditions. Further studies are required to effectively elucidate β‐catenin's contribution to human insulin resistance.

Prior studies have investigated the effect of HFD feeding on β‐catenin levels in non‐muscle tissues. In human adipose tissue, β‐catenin is elevated during obesity relative to lean controls (Chen et al., 2020), and mice fed a HFD exhibit increased β‐catenin expression in colon epithelial cells, as well as colon tumors (Xu et al., 2017). Given the role of β‐catenin in cell proliferation, we may expect to observe differences between adipocytes, intestinal epithelial cells, and muscle cells when exposed to an HFD. Both adipose tissue and epithelial cells exhibit increased cell growth/differentiation during HFD feeding, this likely increases β‐catenin via elevated Wnt‐signaling. In contrast, muscle growth and differentiation are blunted by HFD feeding (Lee et al., 2015; Sitnick et al., 2009) potentially due to decreased β‐catenin levels.

The role of β‐catenin in adipocyte insulin resistance is unclear. Cultured three T3‐L1 adipocytes appear to require β‐catenin for optimal glucose uptake (Dissanayake et al., 2018), and a subpopulation of adipocytes with enhanced Wnt‐signaling positively contribute to glucose handling (Liu et al., 2022). However, adipocytes‐specific deletion of β‐catenin in mice results in improved insulin sensitivity and reduced obesity (Bagchi et al., 2020; Chen et al., 2020). One important distinction is that these studies used a germline knockout model where β‐catenin is deleted from adipocytes for the life of the animal. Because β‐catenin is required for both acute signaling, modulation of the actin cytoskeleton (Masson et al., 2021; Sorrenson et al., 2016), and tissue development, the timing of β‐catenin loss likely impacts the resulting phenotype.

A dual role for β‐catenin in both insulin‐ and exercise‐induced glucose uptake (Masson et al., 2021), coupled with the findings reported here that insulin resistance is associated with lower levels of skeletal muscle β‐catenin is somewhat incongruent with the observation that many insulin‐resistant individuals exhibit normal exercise‐stimulated glucose uptake (Kennedy et al., 1999; King et al., 1993; Kingwell et al., 2002; Wojtaszewski et al., 1999). One potential reconciliation of this point is the distinct protein residues involved in insulin (serine 552) versus exercise‐induced (serine 675) uptake (Masson et al., 2020; Masson et al., 2021). As well as lower β‐catenin levels, HFD‐fed mice had attenuated β‐catenin^S552^ but not Akt^S473^ phosphorylation. This suggests that the remaining β‐catenin in the cell exhibits some form of S552‐specific defect, given that the apparent upstream signaling pathways responsible are functional. Because exercise/contraction acts on β‐catenin via S675 rather than S552, it is possible that the remaining β‐catenin in insulin‐resistant individuals has normal, if not enhanced, S675 functionality. This would then facilitate exercise‐induced glucose uptake despite blunted insulin‐stimulated β‐catenin signaling.

Here, we provide evidence that β‐catenin dysfunction may be involved in the development of skeletal muscle insulin resistance. We build on previous work which identified β‐catenin as a regulator of glucose uptake into muscle via actin remodeling, and the role of actin dysfunction in palmitate‐induced insulin resistance. Finally, we extend the role of β‐catenin into human models of insulin resistance which warrant further investigation.

## AUTHOR CONTRIBUTIONS

S.W.C.M., P.R.S, and T.L.M. conceived and designed research; S.W.C.M., W.C.D., S.C.B., C.P.H., W.M.P., M.G., and D.S.R. performed experiments; S.W.C.M., D.S.R., P.R.S, and T.L.M. interpreted results; S.W.C.M. prepared figures and drafted manuscript; all authors revised and edited manuscript.

## ETHICS STATEMENT

All experiments were approved by the University of Auckland animal ethics committee, Auckland, New Zealand.

## FUNDING INFORMATION

This study was funded by the Rutherford Discovery Fellowship and the University of Auckland Faculty Research Development Fund (all to T.L.M.) and in‐part from a grant from Ministry of Business, Innovation and Employment, NZ [UOOX1404] (to D.S.R).

## CONFLICT OF INTEREST

None.

## Supporting information


Figure S1

Table S1
