# Supplementary figures and images for: A role for β‐catenin in diet‐induced skeletal muscle insulin resistance
Source: Physiol Rep. 2023 Feb 17;11(4):e15536. doi: 10.14814/phy2.15536 (PMC9937784; doi:10.14814/phy2.15536)

Supplementary Figure 1

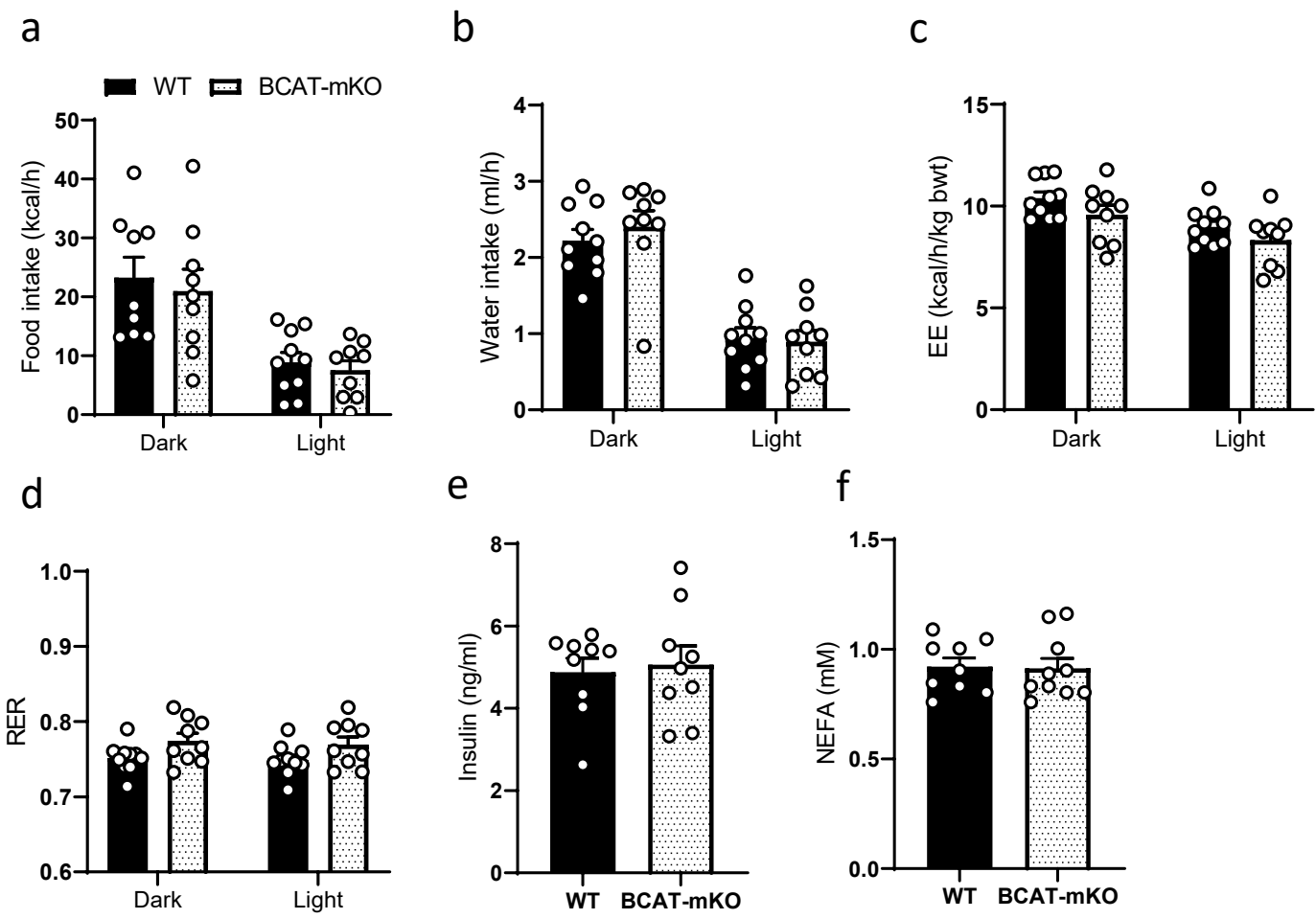

Supplement: Supplementary file 1 — Figure S1 [file PHY2-11-e15536-s001.pdf]
