# Supplementary material for: A role for β‐catenin in diet‐induced skeletal muscle insulin resistance
Source: Physiol Rep. 2023 Feb 17;11(4):e15536. doi: 10.14814/phy2.15536 (PMC9937784; doi:10.14814/phy2.15536)
Supplement: Supplementary file 2 — Table S1 [file PHY2-11-e15536-s002.docx]

**Supplementary Table 1. Immunoblotting antibodies**

| **Protein** | **Supplier** | **Catalogue #** | **Dilution** |
| --- | --- | --- | --- |
| Phospho-S552 Beta-catenin | CST | 9566 | 1:1000 |
| Beta-catenin | Symansis | 3024 | 1:1000 |
| Phospho-S473 Akt | CST | 9271 | 1:1000 |
| Akt 1/2 | CST | 9272 | 1:1000 |
| Αlpha-tubulin | Sigma-Aldrich | T9026 | 1:5000 |
| GAPDH | Abcam | ab9485 | 1:10000 |
| Phospho-S1490 LRP6 | CST | 2568 | 1:1000 |
| LRP6 | CST | 3395 | 1:1000 |
| Phospho-S9 GSK3β | CST | 5558 | 1:1000 |
| GSK3β | CST | 9832 | 1:1000 |
| LRP5 | CST | 5731 | 1:1000 |
| Beta-actin | Sigma-Aldrich | A2228 | 1:10000 |
| Goat anti-Rabbit IgG | Thermofisher | G-21234 | 1:10000 |
| Goat anti-Mouse IgG | Thermofisher | G-21040 | 1:10000 |
